# The association of toll-like receptor 4 gene polymorphisms with primary open angle glaucoma susceptibility: a meta-analysis

**DOI:** 10.1042/BSR20190029

**Published:** 2019-04-02

**Authors:** Narttaya Chaiwiang, Teera Poyomtip

**Affiliations:** Faculty of Optometry, Ramkhamhaeng University, Bangkok 10240, Thailand

**Keywords:** autoimmune disease, Normal tension glaucoma (NTG), Primary Open Angle Glaucoma (POAG), polymorphisms, Toll-like receptor 4 (TLR4)

## Abstract

Primary open angle glaucoma (POAG) and normal tension glaucoma (NTG) cause irreversible blindness while current medications cannot completely inhibit disease progression. An understanding of immunopathogenesis is thus a keystone to develop novel drug targets and genetic markers are still required for early diagnosis. Toll-like receptor 4 (TLR4) is an essential player in inflammation in various diseases. However, the TLR4 polymorphisms have not been completely elucidated in both types of glaucoma. The aim of the present study was to identify the association between TLR4 polymorphism and glaucoma (POAG and NTG) via the use of a comprehensive review and meta-analysis. The relevant studies were collected from PubMed, Excerpta Medica Database (EMBASE), and Web of Science to identify eight included articles, assessed for quality by a modified Newcastle-Ottawa Scale (NOS) for gene association study. A meta-analysis was applied to calculate the pooled odds-ratio and 95% confidence intervals (CIs) to evaluate the association between TLR4 polymorphism and glaucoma. The results revealed that TLR4 rs1927911 A/G, rs12377632 C/T, and rs2149356 G/T significantly decrease the risk of POAG and NTG in allele contrast models 0.71-, 0.71-, and 0.67-fold, respectively. Moreover, rs4986790 A/G and rs4986791 C/T showed a stringent association with POAG in allele contrast, heterozygous, recessive, and overdominant models. In conclusion, this meta-analysis represented a significant correlation between TLR4 polymorphisms and both types of glaucoma suggesting that TLR4 might be involved in the pathogenesis of glaucoma and may be applied as a genetic marker for disease screening.

## Introduction

Glaucoma, characterized by retinal ganglion cell (RGCs) death and optic nerve fiber loss, is a common optic neuropathy which is the second-highest cause of blindness worldwide [1,2]. It was estimated that there will be approximately 79.6 million people living with glaucoma by 2020, increasing to 111 million by 2040. The vast majority of glaucoma types are POAG, which is associated with elevated intraocular pressure (IOP) without a recognizable secondary cause such as ocular surgery, ocular trauma, or laser iridotomy [3,4]. On the other hand, some POAG patients are able to appear normal in terms of IOP of <22 mmHg. This type is classified as NTG. POAG and NTG share similarities in phenotypes composed of having normal anterior chamber angles, peripapillary retinal nerve fiber layer (RNFL) thinning, glaucomatous optic neuropathy (GON), and corresponding visual field (VF) defects. Therefore, it is believed that POAG and NTG illustrate a continuum of open-angle glaucoma in which there is a difference in some causative factors and IOP [5,6]. Although the precise and accurate molecular mechanisms of POAG and NTG have not been investigated, it is possible that patients with POAG and NTG have some overlapping factors in both multiple genetic and environmental interactions.

The emerging roles of inflammation and immunity were suggested as a paradigm shift in glaucoma pathogenesis. A gain-of-function mutation of pro-inflammatory gene TBK1-tumor necrosis factor (TNF) receptor associated factor NF-κB activator (TANK) binding kinase1 associated with NTG [7,8]. In a mouse model of inherited glaucoma, DBA/J2 mice showed a correlation between microglia activation and axon loss [9]. Moreover, early stages of the glaucoma model showed that neuroinflammatory response and inhibition of pro-inflammatory pathways play a role in the neuroprotective effect [10]. Amongst inflammatory molecules, Toll-like receptors (TLRs), referred to as transmembrane pattern recognition receptors (PRRs), play a significant role in innate immunity. The TLR activation initiates the inflammatory process via recognition with pathogen-associated molecular patterns (PAMPs), derived structure from microorganisms known as exogenous ligands, damage-associated molecular patterns (DAMPs), and derived cellular motifs from tissue damage known as endogenous ligands [11]. Currently, there are approximately ten TLRs which are identified in humans. TLR4 was the first to be discovered and is well known through study to bind to lipopolysaccharide (LPS) in Gram-negative bacteria, a lipoprotein of the RNA virus, and several heat shock proteins (HSPs) of host components [12–15]. In recent years, evidence suggests that the TLR4 plays multiple roles which are related to POAG. Activation of TLR4 alters the trabecular meshwork fibrosis during TGF-β stimulation and subsequently causes elevated IOP [16]. TLR4/HMGB1 binding activates RGC apoptosis in the acute glaucoma model [17–19]. The HSPs and LPS, being ligands of TLR4, were previously implicated as candidate antigens of NTG [20]. Moreover, Tenascin-C, another DAMP of TLR4, has been increased in astrocytes of the glaucomatous optic nerve head [21]. Therefore, the TLR4 may play a role in glaucoma pathogenesis.

In this decade, advanced sequencing technology has resulted in personal medicine and biomarker development. To achieve this aim, gene association studies are required as personalized information to predict the disease outcomes. Single nucleotide polymorphisms (SNPs) in the TLR4 gene were associated with both infectious diseases and non-communicable diseases [22–24]. To date, several positions of TLR4 polymorphisms have been examined for proposal as risk or protective factors of glaucoma. However, the results are still controversial, both for POAG and NTG. To solve this, we applied a comprehensive review and meta-analysis between genetic models of TLR4 polymorphism and glaucoma. Our results will provide supporting evidence relating to glaucoma as an autoimmune disease.

## Materials and methods

### Searching strategy

To identify the related article, the searching, dependent on electronic literature databases, was performed in PubMed, EMBASE, and Web of Science. The search terms were updated until 4 December 2018 and used the following keywords: (variants OR genetic polymorphisms OR genetic polymorphism OR genotyping OR SNP OR SNPs OR SNP OR SNPs OR polymorphisms OR polymorphism OR nucleotide polymorphism OR gene variation OR haplotype) AND (TLR-4 OR TLR4 OR receptor, TLR4 OR TLR4 receptor OR toll 4 receptor OR toll-4 receptor OR toll-like receptor 4) AND (POAG OR POAG OR NTG OR NTG). Two independent investiators searched and subsequently screened the titles and abstracts to identify eligible articles based on inclusion and exclusion criteria. The investigator entered into a discussion to resolve disagreements to achieve the final consensus. An additional publication was considered via related article screening.

### Inclusion and exclusion criteria

Selected publications included in our study were: (1) case-control or cohort study on the association of TLR4 polymorphisms and POAG; (2) odds-ratio (OR) or relative risk with a 95% CI; (3) human study, and (4) providing information for all genetic models. The exclusion criteria were: (1) abstract, case-report review, and systematic review; (2) other TLRs study; (3) non-relevant study, and (4) insufficient data.

### Quality assessment

The modified NOS for genetic association study was applied for quality assessment of included articles which were evaluated by two independent investigators [25]. The NOS criteria are separated into three outlines: (1) subject selection; (2) the comparability of subjects; and (3) exposure. The total score is 9, with 0–3 classified as a low-quality study, 4–6 classified as a moderate-quality study, and 7–9 classified as a high-quality study. The moderate-quality and high-quality studies were considered for inclusion in the meta-analysis. Any disagreement was resolved by discussion for any final consensus.

### Data extraction and synthesis

Briefly, the name of the first author, the publication year, the number of cases and controls, genotype distribution of cases and controls, genotyping methods, and case definition were extracted by two investigators independently extracted relevant information from the eligible studies. An external participant was invited as an expert to resolve some disagreements.

### Statistical analysis

To decrease the selection bias, the Hardy-Weinberg equilibrium (HWE) was evaluated by using chi-square testing in control groups with *P*<0.05 showing a deviation from HWE. The strength of association between TLR4 polymorphisms and glaucoma was represented as OR with 95% CIs. All allelic models (allele contrast, homozygous comparison, heterozygous comparison, dominant model, recessive model, and overdominant model) were examined for association by using an adjusted *P*-value for multiple testing via the Bonferroni method. The I^2^ value was considered to assess the heterogeneity amongst different studies. I^2^ <50% and *P*>0.05 was considered a homogeneous population. Consequently, the pool OR was combined using the fixed-effect model otherwise the random-effect models were performed when I^2^ >50% and *P*<0.05. Moreover, publication bias was tested by funnel plot and Egger’s regression test. Sensitivity analysis was considered to evaluate the stability of the meta-analysis result and root of heterogeneity by all studies removing one by one. The statistical analysis and meta-analysis were carried out using MetaGenyo [26].

## Results

### Characteristics of included studies

**Figure 1:**
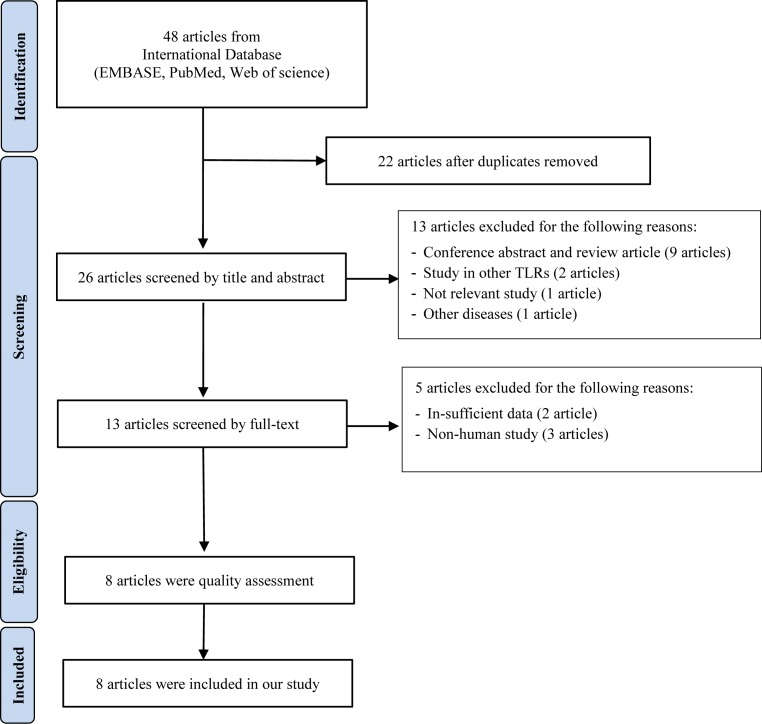
Flow diagram showing review protocol for eligible studies

By using a systematic searching strategy, we identified a total of 48 records relating to POAG from three electronic databases. After removing duplicate articles, 26 studies were enrolled by title and abstract screening and 13 studies were excluded in this step. This resulted in 13 articles being downloaded and the full text being considered. Five articles were excluded. Eventually, eight articles (Figure 1) [27–34], containing ten polymorphisms of TLR4 (rs4986790, rs4986791, rs10759930, rs1927914, rs1927911, rs12377632, rs2149356, rs11536889, rs7037117, and rs7045953), were applied in our quality assessment (Supplementary Table S1) and meta-analysis. Amongst these studies were six articles which studied POAG with IOP >22 mmHg (two articles on rs4986790, two articles on rs4986791, two articles on rs1927911, rs12377632, rs2149356, rs11536889, and one article on rs7037117: Table 1). All the studies of POAG with IOP >22 were based on Mexican, Saudi Arabian, Japanese, and Han Chinese populations. On the other hand, there were three studies conducted on TLR4 polymorphisms (rs10759930, rs1927914, rs1927911, rs12377632, rs2149356, rs11536889, rs7037117, and rs7045953) with NTG of which two studies were carried out with Japanese populations and one study was carried out with a South Korean population.

**Table 1:** General demographics of TLR4 gene polymorphisms and POAG which were included in our study

Authors	Study/ disease setting	Criteria case	Mean case age (years)	Mean control age (years)	Genotyping methods	Positions	NOS
Navarro-Partida et al. 2017 [27]	Mexican population/ POAG	IOP > 22 mmHg in each eye, cup-to-disc ratio >0.7, VF defects determined by Humphrey 24-2 standard automated perimetry and open anterior chamber angle	66.49 ± 14.3	63.28 ± 7.93	Real-time PCR	rs4986790 rs4986791	5
Navarro-Partida et al. 2017 [28]	Mexican population/ POAG	IOP > 22 mmHg in each eye, cup-to-disc ratio >0.7, VF defects determined by Humphrey 24-2 standard automated perimetry and open anterior chamber angle	66.49 ± 14.4	63.28 ± 7.94	Real-time PCR	rs11536889 rs1927911 rs12377632 rs2149356	4
Mousa et al. 2016 [29]	Saudi Arab population/ POAG	Not stated. The author stated that the case participants were clinically confirmed diagnosed	60.90 ± 12.7	57.7 ± 10.4	Real-time PCR	rs4986791	4
Abu-Amero et al. 2017 [30]	Saudi Arab population/ POAG	(1) Appearance of the disc and retina nerve fiber layer, (2) VF abnormalities, and (3) open anterior chamber angles bilaterally on gonioscopy	60.90 ± 12.8	69.7 ± 11.3	Real-time PCR	rs4986790	4
Takano et al. 2012 [31]	Japanese population/ POAG and NTG	(POAG): IOP >22 mmHg in each eye, cup-to-disc ratio >0.7, VF defects determined by Goldmann perimetry and/or Humphrey VF analysis and open anterior chamber angle (NTG): IOP < 22 mmHg, and the same characteristic as that of POAG group. The patients with glaucoma secondary causes were excluded	POAG: 64.60 ± 14.3 NTG: 58.60 ± 13.1	57.7 ± 10.6	PCR-sequencing	A	4
Chen et al. 2012 [32]	Chinese population/ POAG	(1) Shaffer grade III or IV open iridocorneal angle on gonioscopy, (2) Humphrey VF analysis, and (3) IOP ≥22 mmHg was measured by applanation tonometry	48.57 ± 17.5	72.2 ± 6.7	Real-time PCR	rs7037117	5
Suh et al. 2011 [33]	South Korean population/ NTG	IOP <24 mmHg in each eye was measured by Goldmann tonometer, VF defects determined by Humphrey 30-2 standard automated perimetry in association with an open angle on gonioscopy. These are not history of angle closure, ocular trauma, corneal opacity, laser iridotomy, inflammatory eye disease, or ocular surgery	NA	NA	Real-time PCR	A	6
Shibuya et al. 2008 [34]	Japanese population/ NTG	NTG: (1) the presence of GON with corresponding VF loss, (2) normal open angle with angle width of Shaffer grade 2 or higher and (3) IOP <22 mmHg on repeat measurement with Goldmann applanation tonometry	NA	NA	Real-time PCR	A	5

A represents TLR4 polymorphism at rs10759930, rs1927914, rs1927911, rs12377632, rs2149356, rs11536889, rs7037117, and rs7045953

All studies were of case-control design and published between 2008 and 2017. The case definition of POAG was based on clinical manifestation. Briefly, the patient has VF defects or abnormalities, no history of angle closure or secondary causes. Only one study in NTG applied IOP <24 mmHg which has a difference from two studies (IOP < 22 mmHg) (Table 1).

One study performed genotyping using PCR-sequencing and eight studies assessed using real-time PCR. The genotype details are presented in Table 2. The genotype distribution of control in all studies was in accordance with the Hardy-Weinberg equilibrium. Moreover, we also showed the minor allele frequency (MAF) for which nine polymorphisms represented the same minor allele except for rs12377632 for which there was one study which showed a different result (Table 2).

**Table 2:** The genotype distribution of TLR4 polymorphisms in cases and controls

Authors	Allele (1/2)	Cases			Controls			HWE *P*-value	MAF	
		11	12	22	11	12	22		Cases	Controls
rs4986790	A/**G**									
Navarro-Partida et al. 2017 [27]		166	20	1	106	3	0	0.88	0.06	0.01
Abu-Amero et al. 2017 [30]		70	14	1	86	8	1	0.13	0.09	0.05
rs4986791	C/**T**									
Navarro-Partida et al. 2017 [27]		165	21	1	105	4	0	0.85	0.06	0.02
Mousa et al. 2016 [29]		73	11	1	87	8	0	0.67	0.08	0.04
rs10759930	T/**C**									
Takano et al. 2012 [31]		190	262	97	103	85	28	0.12	0.42	0.33
Suh et al. 2011 [33]		52	72	23	126	191	63	0.51	0.40	0.42
Shibuya et al. 2008 [34]		81	127	42	137	141	40	0.69	0.42	0.35
rs1927914	A/**G**									
Takano et al. 2012 [31]		184	270	95	105	82	29	0.05	0.42	0.32
Suh et al. 2011 [33]		52	72	23	126	192	62	0.44	0.4	0.42
Shibuya et al. 2008 [34]		82	126	42	137	141	40	0.69	0.42	0.35
rs1927911	G/**A**									
Navarro-Partida et al. 2017 [28]		83	87	17	64	34	11	0.06	0.32	0.26
Takano et al. 2012 [31]		190	267	92	106	85	25	0.22	0.41	0.31
Suh et al. 2011 [33]		53	71	23	129	190	61	0.52	0.40	0.41
Shibuya et al. 2008 [34]		87	122	41	141	135	42	0.29	0.41	0.34
rs12377632	**C/T**									
Navarro-Partida et al. 2017 [28]		27	89	71	27	51	31	0.51	0.62	0.52
Takano et al. 2012 [31]		190	280	79	104	87	25	0.30	0.40	0.32
Suh et al. 2011 [33]		54	70	23	127	191	62	0.49	0.39	0.41
Shibuya et al. 2008 [34]		86	122	42	140	138	40	0.51	0.41	0.34
rs2149356	G/**T**									
Navarro-Partida et al. 2017 [28]		61	100	26	61	36	12	0.07	0.41	0.28
Takano et al. 2012 [31]		192	262	95	107	85	24	0.26	0.41	0.31
Suh et al. 2011 [33]		54	70	23	128	191	61	0.46	0.39	0.41
Shibuya et al. 2008 [34]		87	122	41	140	138	40	0.51	0.41	0.34
rs11536889	G/**C**									
Navarro-Partida et al. 2017 [28]		146	40	1	84	22	3	0.30	0.11	0.13
Takano et al. 2012 [31]		291	228	30	127	76	13	0.72	0.26	0.24
Suh et al. 2011 [33]		77	62	8	221	139	20	0.76	0.27	0.24
Shibuya et al. 2008 [34]		146	93	11	177	119	22	0.74	0.23	0.26
rs7037117	A/**G**									
Takano et al. 2012 [31]		333	189	27	153	54	9	0.14	0.22	0.17
Chen et al. 2012 [32]		124	46	14	145	77	8	0.57	0.20	0.20
Suh et al. 2011 [33]		85	51	11	211	143	26	0.79	0.25	0.26
Shibuya et al. 2008 [34]		138	98	14	213	94	11	0.87	0.25	0.18
rs7045953	A/**G**									
Takano et al. 2012 [31]		465	81	3	191	24	1	0.79	0.08	0.06
Suh et al. 2011 [33]		126	19	2	314	60	6	0.12	0.08	0.09
Shibuya et al. 2008 [34]		203	45	2	269	49	0	0.14	0.10	0.08

Bold text indicates minor allele.

### Meta-analysis results and publication bias

To investigate the potential association of other TLR4 polymorphisms with glaucoma (POAG and NTG), three studies about rs10759930 polymorphism (946 cases and 914 controls), three studies about rs1927914 polymorphism (946 cases and 914 controls), four studies about rs1927911 polymorphism (1133 cases and 1023 controls), four studies about rs12377632 polymorphism (1133 cases and 1023 controls), four studies about rs2149356 polymorphism (1133 cases and 1023 controls), four studies about rs11536889 polymorphism (1133 cases and 1023 controls), four studies about rs7037117 polymorphism (1130 cases and 1144 controls), and three studies about rs7045953 polymorphism (946 cases and 914 controls) were enrolled for meta-analysis resulting in three positions (rs1927911, rs12377632, and rs2149356) showing significant association with glaucoma (Table 3) in five genetic models, three genetic models, and five genetic models, respectively. The allele contrast model, homozygous model, and recessive model were associated with glaucoma in all positions (allele contrast model, rs1927911; OR = 0.78, *P*=0.02, rs12377632; OR = 0.78, *P*=0.02, rs2149356; OR = 0.71, *P*=0.02, homozygous model, rs1927911; OR = 0.68, *P*=0.01, rs12377632; OR = 0.65, *P*=0.00, rs2149356; OR = 0.62, *P*=0.02, recessive model, rs1927911; OR = 0.69, *P*=0.02, rs12377632; OR = 0.70, *P*=0.04, rs2149356; OR = 0.64, *P*=0.03). An Egger’s test and funnel plot suggested that no publication bias exists in these genetic models (Supplementary Table S1 and Supplementary Figure S1). Altogether, the results suggest that the TLR4 polymorphism (rs1927911, rs12377632, and rs2149356) is related to glaucoma in both POAG and NTG.

**Table 3:** The meta-analysis of TLR4 polymorphisms and glaucoma (POAG and NTG)

SNPs 1/2	Genetic models						
	A	B	C	D	E	F	G
rs10759930 T/C							
OR (95% CI)	*0.80 (0.62;1.04)*	0.68 (0.43;1.07)	0.91 (0.68;1.22)	0.74 (0.52;1.04)	0.79 (0.60;1.04)	0.73 (0.50;1.05)	*1.23 (1.00;1.50)*
*p-value*^a^	*0.099*	0.0969	0.5602	0.0917	0.0972	0.0946	*0.0395*
I^2^(%)	*69.49*	55.13	0.00	61.23	0.00	69.73	*21.27*
*p-value*^b^	*0.03*	0.10	0.87	0.07	0.40	0.04	*0.28*
rs1927914 A/G							
OR (95% CI)	*0.80 (0.61;1.04)*	0.68 (0.44;1.05)	0.95 (0.71;1.27)	*0.72 (0.48;1.07)*	0.80 (0.61;1.06)	*0.71 (0.47;1.07)*	1.25 (0.94;1.67)
*p-value*^a^	*0.0957*	0.0845	0.7305	*0.1117*	0.1248	*0.0002*	0.1201
I^2^ (%)	*70.85*	52.25	0.00	*71.07*	0.00	*74.80*	52.19
*p-value*^b^	*0.03*	0.12	0.86	*0.03*	0.50	*0.00*	0.12
rs1927911 G/A							
OR (95% CI)	**0.78 (0.63;0.97)**	**0.68 (0.52;0.91)**	*0.98 (0.74;1.30)*	**0.69 (0.50;0.94)**	0.82 (0.63;1.09)	**0.69 (0.51;0.94)**	**1.32 (1.10;1.59)**
*p-value*^a^	**0.0227**	**0.0095**	*0.8788*	**0.0186**	0.147	**0.0169**	**0.0033**
I^2^ (%)	**57.17**	**34.69**	*0.00*	**58.89**	0.00	**61.98**	**47.70**
*p-value*^b^	**0.07**	**0.20**	*0.62*	**0.06**	0.51	**0.04**	**0.13**
rs12377632 C/T							
OR (95% CI)	**0.78 (0.63;0.97)**	**0.65 (0.49;0.85)**	0.90 (0.69;1.17)	0.72 (0.52;1.00)	0.78 (0.61;1.00)	**0.70 (0.50;0.98)**	1.20 (1.00;1.45)
*p-value*^a^	**0.0245**	**0.0024**	0.4159	0.3675	0.0471	**0.0397**	0.0442
I^2^ (%)	**59.28**	**44.68**	0.00	59.31	0.00	**66.11**	40.13
*p-value*^b^	**0.06**	**0.14**	0.86	0.06	0.60	**0.03**	0.17
rs2149356 G/T							
OR (95% CI)	**0.74 (0.57;0.96)**	**0.62 (0.41;0.92)**	*0.91 (0.69;1.20)*	**0.65 (0.43;0.99)**	**0.76 (0.58;0.98)**	**0.64 (0.43;0.96)**	1.35 (0.97;1.88)
*p-value*^a^	**0.0241**	**0.0229**	*0.4955*	**0.0425**	**0.0371**	**0.0309**	0.0747
I^2^ (%)	**72.99**	**50.68**	*0.00*	**76.15**	**0.00**	**77.88**	67.73
*p-value*^b^	**0.01**	**0.10**	*0.75*	**0.00**	**0.51**	**0.00**	0.02
rs11536889 G/C							
OR (95% CI)	0.97 (0.83;1.13)	1.20 (0.78;1.84)	1.40 (0.90;2.16)	0.87 (0.72;1.06)	1.27 (0.84;1.94)	0.91 (0.75;1.10)	1.17 (0.96;1.41)
*p-value*^a^	0.7054	0.4045	0.1351	0.1599	0.594	0.3168	0.1153
I^2^ (%)	11.33	3.39	0.00	0.00	0.00	0.00	0.00
*p-value*^b^	0.33	0.38	0.64	0.54	0.48	0.39	0.66
rs7037117 A/G							
OR (95% CI)	0.83 (0.65;1.04)	0.67 (0.44;1.00)	0.76 (0.50;1.16)	*0.88 (0.58;1.32)*	*0.70 (0.47;1.04)*	*0.84 (0.59;1.18)*	*1.11 (0.74;1.66)*
*p-value*^a^	0.1116	0.0476	0.2056	*0.5233*	*0.0752*	*0.311*	*0.6291*
I^2^ (%)	56.82	0.00	23.83	*77.33*	*0.00*	*72.32*	*78.02*
*p-value*^b^	0.07	0.62	0.27	*0.00*	*0.59*	*0.01*	*0.00*
rs7045953 A/G							
OR (95% CI)	0.87 (0.67;1.13)	0.78 (0.23;2.60)	0.76 (0.22;2.64)	0.88 (0.66;1.17)	0.78 (0.23;2.59)	0.87 (0.66;1.15)	1.13 (0.85;1.50)
*p-value*^a^	0.2853	0.6812	0.6672	0.3715	0.6789	0.3194	0.3852
I^2^ (%)	27.21	0.00	0.00	16.26	0.00	23.98	14.22
*p-value*^b^	0.25	0.50	0.60	0.30	0.52	0.27	0.31

a OR p-value, ^b^ heterogeneous p-value

A: Allele contrast model, B: Homozygous model, C: Heterozygous (12 vs. 22), D: Heterozygous (11 vs. 12), E: Dominant model, F: Recessive model, and G: Overdominant model.

Bold text showed statistical significance in meta-analysis model. Italic text represented Egger’s test *P-value* < 0.05.

### Subgroup analysis of POAG and NTG

**Figure 2:**
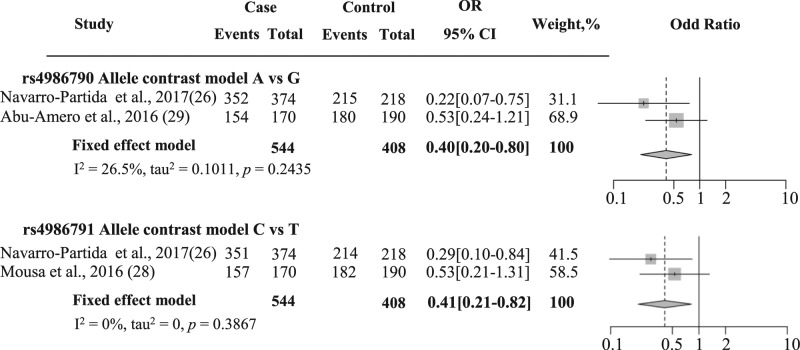
Forest plot of association between two TLR4 polymorphisms and POAG The area of the square was proportional to the study’s weight. The horizontal line represents 95% CI. The overall effect was illustrated as diamonds with the lateral points showing the CI. The forest plots of rs4986790 and rs4986791 associated with POAG were shown as an allele contrast model.

Due to limitations of materials of NTG in TLR4 rs4986790 and rs4986791 studies, there were two studies in POAG which were combined by a meta-analysis that included 272 cases and 204 healthy controls (Table 4). The meta-analysis results showed that four genetic models were statistically related between these positions and the susceptibility of POAG: allele contrast model (rs4986790; OR = 0.40, *P* =0.01, rs4986791; OR = 0.41, *P*=0.01) (Figure 2), heterozygous model (rs4986790; OR = 0.36, *P*=0.01, rs4986791; OR = 0.44, *P*=0.03), recessive model (rs4986790; OR = 0.37, *P*=0.01, rs4986791; OR = 0.42, *P*=0.02), and overdominant model (rs4986790; OR = 2.73, *P* =0.01, rs4986791; OR = 2.21, *P* =0.03) (Table 4).

**Table 4:** Meta-analysis of rs4986791 and rs4986790 associated with POAG

Genetic models	OR (95% CI)	*p-value*^a^	I^2^(%)	*p-value*^b^
rs4986791				
Allele contrast C vs. T	0.41 (0.20;0.82)	0.0117	0.00	0.38
Homozygote compairison CC vs. TT	0.38 (0.04;3.71)	0.4074	0.00	0.79
Heterozygote comparison CT vs. TT	1.84 (0.08;8.92)	0.8861	0.00	0.6
Heterozygote comparison CC vs. CT	0.44 (0.21;0.92)	0.0294	0.00	0.34
Dominant model CC+CT vs. TT	0.41 (0.04;3.97)	0.4408	0.00	0.78
Recessive model CC vs. CT + TT	0.42 (0.20;0.86)	0.0173	0.00	0.36
Overdominant model CT vs. CC+TT	2.21 (1.07;4.56)	0.0315	0.00	0.33
rs4986790				
Allele contrast A vs. G	0.40 (0.20;0.80)	0.0096	26.49	0.24
Homozygote comparison AA vs. GG	0.67 (0.08;0.51)	0.7112	0.00	0.84
Heterozygote comparison AG vs. GG	1.83 (0.20;16.67)	0.5905	0.00	0.96
Heterozygote comparison AA vs. AG	0.36 (0.17;0.76)	0.0075	0.00	0.38
Dominant model AA+AG vs. GG	0.73 (0.08;6.03)	0.7745	0.00	0.83
Recessive model AA vs. AG + GG	0.37 (0.18;0.76)	0.0074	1.54	0.31
Overdominant model AG vs. AA+GG	2.73 (1.30;5.73)	0.0077	0.00	0.38

a OR *P*-value, ^b^ heterogeneous *P*-value.

Previous results in combined glaucoma types showed great heterogeneity amongst studies relating to rs1927911, rs12377632, and rs2149356 (I^2^ > 50%, *P*<0.05). Therefore, we employed stratification by using the glaucoma types (POAG and NTG). All positions were associated with POAG in the recessive model (rs1927911; OR = 0.60, *P*=0.00 (Table 5), rs12377632; OR = 0.60, *P*=0.01 (Table 6), rs2149356; OR = 0.51, *P*=0.03 (Table 7). However, the rs1927911 correlated with NTG in the homozygous model (OR = 0.70, *P*=0.025 (Table 5)).

**Table 5:** Meta-analysis result of rs1927911 based on subgroup analysis

SNPs	OR (95% CI)	*P*-value	Heterogeneity		Effect models	Egger’s *P*-value
			I^2^ (%)	*P*-value		
Allele contrast						
POAG	0.58 (0.61–1.00)	0.0538	0	0.58	F	-
NTG	0.82 (0.65–1.04)	0.0969	59.27	0.09	R	0.329
Homozygous model						
POAG	0.79 (0.46–1.35)	0.3891	0	0.85	F	-
**NTG**	**0.70 (0.51–0.95)**	**0.0258**	**38.59**	**0.19**	**F**	**0.37**
Heterozygous model (GA vs. AA)						
POAG	1.35 (0.80–2.28)	0.2574	0	0.56	F	-
NTG	0.91 (0.67–1.23)	0.5256	0	0.88	F	0.95
Heterozygous model (GG vs. GA)						
**POAG**	**0.56 (0.39–0.81)**	**0.0023**	**0**	**0.5618**	**F**	-
NTG	0.77 (0.62–0.96)	0.0208	46.34	0.15	F	0.03
Dominant model						
POAG	1.05 (0.64–1.71)	0.8558	0	0.8274	F	-
NTG	0.80 (0.60–1.07)	0.1329	0	0.49	F	0.68
Recessive model						
**POAG**	**0.60 (0.43–0.86)**	**0.0047**	**0**	**0.64**	**F**	**-**
NTG	0.77 (0.56–1.05)	0.1024	58.24	0.09	R	0.0437
Overdominant model						
**POAG**	**1.64 (1.17–2.33)**	**0.0042**	**0**	**0.40**	**F**	**-**
NTG	1.18 (0.96–1.44)	0.1139	0	0.37	F	0.11

Bold text showed statistical significance in meta-analysis model.

**Table 6:** Meta-analysis result of rs12377632 based on subgroup analysis

SNPs	OR (95% CI)	*P*-value	Heterogeneity		Effect models	Egger’s *P*-value
			I^2^ (%)	*P-value*		
Allele contrast						
**POAG**	**0.71 (0.56–0.90)**	**0.0050**	**0**	**0.60**	**F**	-
NTG	0.86 (0.70–1.07)	0.1807	54.03	0.11	R	0.27
Homozygous model						
**POAG**	**0.51 (0.32–0.83)**	**0.0062**	**0**	**0.52**	**F**	-
NTG	0.81 (0.60–1.11)	0.1932	33.83	0.22	F	0.18
Heterozygous model (CT vs. TT)						
POAG	0.80 (0.53–1.21)	0.2875	0	0.80	F	-
NTG	1.03 (0.76–1.41)	0.8091	0	0.37	F	0.35
Heterozygous model (CC vs. CT)						
**POAG**	**0.65 (0.43–0.97)**	**0.0365**	**0**	**0.63**	**F**	-
NTG	0.77 (0.53–1.12)	0.1787	65.02	0.05	R	0.12
Dominant model						
POAG	0.68 (0.46–1.01)	0.0564	0	0.10	F	-
NTG	0.92 (0.69–1.23)	0.589	0	0.38	F	0.03
Recessive model						
**POAG**	**0.60 (0.41–0.88)**	**0.0091**	**0**	**0.5**	**F**	-
NTG	0.78 (0.55–1.11)	0.1701	64.95	0.06	R	0.01
Overdominant model						
POAG	1.10 (0.79–1.54)	0.5772	0	0.71	F	-
NTG	1.23 (0.90–1.69)	0.2082	59.83	0.08	R	0.43

Bold text showed statistical significance in meta-analysis model.

**Table 7:** Meta-analysis result of rs2149356 based on subgroup analysis

SNPs	OR (95% CI)	*P-value*	Heterogeneity		Effect models	Egges’s *P-value*
			I^2^ (%)	*P-value*		
Allele contrast						
POAG	0.69 (0.46–1.03)	0.0716	63.21	0.10	R	-
NTG	0.82 (0.63–1.06)	0.1260	67.34	0.05	R	0.36
Homozygous model						
POAG	0.61 (0.36–1.02)	0.0596	0	0.34	F	-
NTG	0.69 (0.44–1.09)	0.111	53.44	0.12	R	0.40
Heterozygous model (GT vs. TT)						
POAG	1.18 (0.72–1.97)	0.4993	0	0.80	F	-
NTG	0.85 (0.63–1.16)	0.3116	0	0.8	F	0.9
Heterozygous model (GG vs. GT)						
**POAG**	**0.49 (0.34–0.71)**	**0.0243**	**63.57**	**0.1**	**R**	**-**
NTG	0.80 (0.58–1.11)	0.1811	52.76	0.12	R	0.07
Dominant model						
POAG	0.88 (0.55–1.41)	0.5936	0	0.64	F	-
NTG	0.77 (0.57–1.02)	0.0695	2.26	0.36	F	0.69
Recessive model						
**POAG**	**0.51 (0.28–0.93)**	**0.0277**	**65.20**	**0.09**	**R**	**-**
NTG	0.78 (0.55–1.10)	0.1552	65.02	0.06	R	0.07
Overdominant model						
**POAG**	**1.76 (1.03–3.02)**	**0.0391**	**59.76**	**0.11**	**R**	**-**
NTG	1.14 (0.93–1.39)	0.2054	2.94	0.36	F	0.15

Bold text showed statistical significance in meta-analysis model.

**Figure 3:**
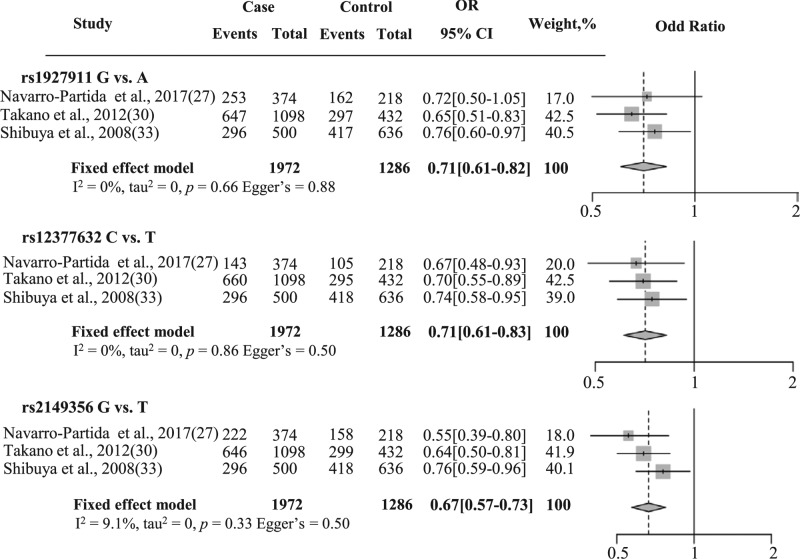
Forest plot of association between two TLR4 polymorphisms and NTG The area of the square was proportional to the study’s weight. The horizontal line represents 95% CI. The overall effect was illustrated as diamonds with the lateral points showing the CI. The forest plots of rs1927911, rs12377632, and rs214356 associated with NTG were shown as an allele contrast model.

Since the stratification by using the glaucoma types still showed heterogeneity, we conducted a sensitivity analysis to reveal the influence of each study on the pooled OR of glaucoma. The sensitivity analysis of the association between SNPs (rs1927911, rs12377632, and rs2149356) and glaucoma was performed in the allele contrast model and shown in Table 8. The result suggested that there is one study which is the root of heterogeneity which affected the pooled OR. Finally, the source of heterogeneity was excluded from the meta-analysis resulting in TLR4 polymorphism association with glaucoma (Figure 3).

**Table 8:** Sensitivity analysis for the allele contrast model in the meta-analysis

Omitting study	OR	95% CI	I^2^	*P*-value
rs1927911				
Navarro-Partida et al. 2017a	0.80	(0.61;1.04)	70.74	0.03
Takano et al. 2012	0.84	(0.67;1.06)	48.09	0.14
Suh et al. 2011	0.71	(0.61;0.82)	0.00	0.66
Shibuya et al. 2008	0.79	(0.58;1.08)	71.29	0.03
overall effect	0.78	(0.64;0.97)	57.17	0.07
rs12377632				
Navarro-Partida et al. 2017a	0.82	(0.63;1.06)	68.36	0.04
Takano et al. 2012	0.82	(0.62;1.09)	67.12	0.04
Suh et al. 2011	0.71	(0.61;0.83)	0.00	0.86
Shibuya et al. 2008	0.80	(0.59;1.09)	71.92	0.03
overall effect	0.79	(0.64;0.97)	72.99	0.01
rs2149356				
Navarro-Partida et al. 2017a	0.80	(0.60;1.07)	75.42	0.02
Takano et al. 2012	0.78	(0.55;1.10)	76.64	0.01
Suh et al. 2011	0.66	(0.56;0.78)	9.06	0.33
Shibuya et al. 2008	0.73	(0.49;1.08)	81.96	0.00
overall effect	0.74	(0.57;0.96)	72.99	0.01

## Discussion

Our meta-analysis was based on systematic collected studies relating to TLR4 gene polymorphisms and glaucoma. To our knowledge, this is the first study that attempted to combine the POAG and NTG studies because these two types of glaucoma are overlapping in some factors [5,6]. Interestingly, combined analysis showed rs1927911, rs12377632, and rs2149356 in the allele contrast model were strongly associated with glaucoma (POAG and NTG) suggesting that the POAG and NTG share some pathogenesis factors that may relate to TLR4. Additionally, the different MAS in rs12377632 did not disturb the meta-analysis result [28] suggesting that there is a different population structure in this position [35]. This was supported by stringent heterogeneity. To eliminate this limitation, the sensitivity analysis was applied and illustrated a similar result after removing one publication in NTG, which is a source of heterogeneity. It is possible that Suh et al. [33] performed in a different setting and the Korean ethnic group may have some factors which interfere with the result.

Besides the combined analysis, we conducted a subgroup analysis by using types of diseases (POAG or NTG). Surprisingly, rs1927911, rs12377632, and rs2149356, which exist in an intron, were still associated with POAG. However, the functions of polymorphisms have not been examined. Only rs1927911 was associated with NTG. It is possible that there are functions relating to RNA stability and regulation, resulting in alteration of protein expression because several translated regions or a part of intron might relate to miRNA [36]. Moreover, previous *in silico* analysis showed that rs2149356G is able to form miRNA which targets autophagy genes [37]. Therefore, rs1927911 and rs12377632 should be under this circumstance. Definitely, all positions should be validated by *in vitro* functional assay to prove this hypothesis.

Additionally, coding polymorphisms of TLR4 rs4986790 A/G (Asp299Gly) and rs4986791 C/T (Thr399Ile), for which both mutations exist in a co-segregation, were also robustly associated with POAG. Taken together, this implied that the TLR4 might play an essential role in POAG pathogenesis. Although the exact functions of these mutations are underinvestigated, there is evidence indicating that these mutations enhance cell death in hepatic stellate cells [38].

Moreover, Asp299Gly and Thr399Ile impair the ability to respond to TLR4 ligands in colorectal cancer cell lines [39]. On the contrary, crystallography studies show that it did not intrude upon LPS binding [40]. Therefore, these mutations may cause RGC apoptosis in POAG, by which several endogenous ligands of TLR4 were up-regulated in the glaucomatous eye [41].

TLR4 activation plays a role in innate immunity and subsequently the adaptive immune response [42]. Currently, several lines of evidence showed that TLR4 relates to the pathophysiology of various diseases as well as autoimmunity [43]. For instance, TLR4 knockout mice reduce autoantibody production and vasculature inflammation in systemic lupus erythematosus (SLE) and atherosclerosis, respectively [44,45]. The activated TLR4 by using endogenous ligands induces pro-inflammatory cytokine and chemokine production in both human synovial fibroblasts and peripheral blood mononuclear cells (PBMC) from rheumatoid arthritis patients leading to cartilage inflammation and degeneration [46–48]. In multiple sclerosis patients and experimental autoimmune encephalomyelitis models, the TLR4 was up-regulated and HMGB-1, a TLR4 ligand, plays a role in the disease progression [49–51]. Additionally, TLR4 polymorphisms are associated with various inflammatory diseases such as aortic aneurysmal disease, periodontitis, psoriasis arthritis, and Crohn’s disease [52–55].

In the recent year, there is new evidence suggesting that CD4^+^ T cells enter into the retina and cause neurodegeneration in the glaucoma model. The CD4^+^ T cells are primed by HSP of normal flora and subsequently crossreacted with mouse or human HSP, TLR4 ligands [56]. Moreover, increasing oral microflora is correlated with microglial activation and neuronal loss via TLR4 signaling, while inhibition of TLR4 causes the neuroprotective effect [57]. In addition, several previous studies indicated that glaucoma (POAG and NTG) has a paradigm shift into inflammatory disease and autoimmunity [21,58–60]. However, to support this paradigm, further evidence is still required. Our study reported that the TLR4 polymorphisms are associated with POAG and NTG which serve as supporting material for glaucoma relating to inflammatory disease and autoimmunity.

There are limitations which appeared in this meta-analysis and these should not be ignored to improve validity and reliability. First, the sample size should be expanded. Second, a relative descent is biased in the Japanese population. The other population descents are still required, especially for the African, Caucasian, and Han Chinese populations. Moreover, additional factors may affect the results, such as co-segregation events whereby our meta-analysis cannot assess the haplotype between TLR4 polymorphisms and glaucoma. At last, the natural history of IOP is not simplified by a single measurement. However, several included articles, which were enrolled in our meta-analysis, did not mention in this regard. It is well known that the IOP is fluctuation over 24 h [61]. Therefore, this may decrease the power of this meta-analysis.

In conclusion, the present study demonstrated that there are associations between TLR4 rs1927911, rs12377632, and rs2149356 and glaucoma (POAG and NTG), while rs4986790 A/G and rs4986791 C/T strongly decrease the risk of POAG suggesting that TLR4 may play a role in glaucoma pathogenesis, which should be classified as a neuroinflammatory and autoimmune disease, and should be considered as a genetic marker for POAG. Previously, the role of TLR4 in POAG was exploited and suggested that it may save as a potential therapeutic strategy [62]. However, the functions of TLR4 polymorphisms in the glaucoma model are unidentified. Therefore, to confirm these associations, the laboratory experiment and a well-designed case-control study are still required for the authentication of the results.

## Data availability

The data for meta-analysis are available in Table 2. The other data are available from the corresponding author upon request.

## Supporting information

**Supplementary Figure S1:** 

**Supplement Table 1:** The NOS analysis of inclued studies

**Supplement Table 2:** The Egger’s test showed publication bias of meta-analysis in Table 3

## Abbreviations

DAMPs: damage-associated molecular patterns
EMBASE: Excerpta Medica Database
GON: glaucomatous optic neuropathy
HSP: heat shock protein
HWE: Hardy-Weinberg equilibrium
IOP: intraocular pressure
LPS: lipopolysaccharide
MAF: minor allele frequency
NOS: Newcastle-Ottawa scale
NTG: normal tension glaucoma
POAG: primary open-angle glaucoma
RGC: retinal ganglion cell
SNP: single nucleotide polymorphism
TLR: toll-like receptor
TNF: tumor necrosis factor
VF: visual field

## References

[1] Hood D.C. (2017) Improving our understanding, and detection, of glaucomatous damage: an approach based upon optical coherence tomography (OCT). Prog. Retin. Eye. Res. 57, 46–75 10.1016/j.preteyeres.2016.12.002 28012881 PMC5350042

[2] Quigley H.A. (1996) Number of people with glaucoma worldwide. Br. J. Ophthalmol. 80, 389–393 10.1136/bjo.80.5.389 8695555 PMC505485

[3] Tham Y.C., Li X., Wong T.Y., Quigley H.A., Aung T. and Cheng C.Y. (2014) Global prevalence of glaucoma and projections of glaucoma burden through 2040: a systematic review and meta-analysis. Ophthalmology 121, 2081–2090 10.1016/j.ophtha.2014.05.013 24974815

[4] Quigley H.A. and Broman A.T. (2006) The number of people with glaucoma worldwide in 2010 and 2020. Br. J. Ophthalmol. 90, 262–267 10.1136/bjo.2005.081224 16488940 PMC1856963

[5] Carr-Lopez S.M., Shek A., Lastimosa J., Patel R.A., Woelfel J.A., Galal S.M. (2014) Medication adherence behaviors of Medicare beneficiaries. Patient Prefer Adherence 8, 1277–1284 10.2147/PPA.S64825 25258521 PMC4172241

[6] Esporcatte B.L. and Tavares I.M. (2016) Normal-tension glaucoma: an update. Arq. Bras. Oftalmol. 79, 270–276 10.5935/0004-2749.20160077 27626157

[7] Fingert J.H., Robin A.L., Stone J.L., Roos B.R., Davis L.K., Scheetz T.E. (2011) Copy number variations on chromosome 12q14 in patients with normal tension glaucoma. Hum. Mol. Genet. 20, 2482–2494 10.1093/hmg/ddr123 21447600 PMC3098731

[8] Ahmad L., Zhang S.Y., Casanova J.L. and Sancho-Shimizu V. (2016) Human TBK1: a Gatekeeper of Neuroinflammation. Trends Mol. Med. 22, 511–527 10.1016/j.molmed.2016.04.006 27211305 PMC4890605

[9] Bosco A., Breen K.T., Anderson S.R., Steele M.R., Calkins D.J. and Vetter M.L. (2016) Glial coverage in the optic nerve expands in proportion to optic axon loss in chronic mouse glaucoma. Exp. Eye. Res. 150, 34–43 10.1016/j.exer.2016.01.014 26851485 PMC4972706

[10] Soto I. and Howell G.R. (2014) The complex role of neuroinflammation in glaucoma. Cold Spring Harb. Perspect. Med. 4, 10.1101/cshperspect.a017269 24993677 PMC4109578

[11] Tang D., Kang R., Coyne C.B., Zeh H.J. and Lotze M.T. (2012) PAMPs and DAMPs: signal 0s that spur autophagy and immunity. Immunol. Rev. 249, 158–175 10.1111/j.1600-065X.2012.01146.x 22889221 PMC3662247

[12] Modhiran N., Watterson D., Blumenthal A., Baxter A.G., Young P.R. and Stacey K.J. (2017) Dengue virus NS1 protein activates immune cells via TLR4 but not TLR2 or TLR6. Immunol. Cell Biol. 95, 491–495 10.1038/icb.2017.5 28220810

[13] Fang H., Wu Y., Huang X., Wang W., Ang B., Cao X. (2011) Toll-like receptor 4 (TLR4) is essential for Hsp70-like protein 1 (HSP70L1) to activate dendritic cells and induce Th1 response. J. Biol. Chem. 286, 30393–30400 10.1074/jbc.M111.266528 21730052 PMC3162398

[14] Asea A., Rehli M., Kabingu E., Boch J.A., Bare O., Auron P.E. (2002) Novel signal transduction pathway utilized by extracellular HSP70: role of toll-like receptor (TLR) 2 and TLR4. J. Biol. Chem. 277, 15028–15034 10.1074/jbc.M200497200 11836257

[15] Cochet F. and Peri F. (2017) The role of carbohydrates in the lipopolysaccharide (LPS)/toll-like receptor 4 (TLR4) Signalling. Int. J. Mol. Sci. 18, 10.3390/ijms18112318 29099761 PMC5713287

[16] Hernandez H., Medina-Ortiz W.E., Luan T., Clark A.F. and McDowell C.M. (2017) Crosstalk between transforming growth factor beta-2 and toll-like receptor 4 in the trabecular meshwork. Invest Ophthalmol. Vis. Sci. 58, 1811–1823 10.1167/iovs.16-21331 28346614 PMC5374883

[17] Chi W., Li F., Chen H., Wang Y., Zhu Y., Yang X. (2014) Caspase-8 promotes NLRP1/NLRP3 inflammasome activation and IL-1beta production in acute glaucoma. Proc. Natl Acad. Sci. U.S.A. 111, 11181–11186 10.1073/pnas.1402819111 25024200 PMC4121847

[18] Chi W., Chen H., Li F., Zhu Y., Yin W. and Zhuo Y. (2015) HMGB1 promotes the activation of NLRP3 and caspase-8 inflammasomes via NF-kappaB pathway in acute glaucoma. J. Neuroinflamm. 12, 137 10.1186/s12974-015-0360-2 26224068 PMC4518626

[19] Sun S., He M., VanPatten S. and Al-Abed Y. (2018) Mechanistic insights into high mobility group box-1 (HMGb1) induced toll-like receptor 4 (TLR4) dimer formation. J. Biomol. Struct. Dyn. 1–30 10.1080/07391102.2018.1526712 [Epub ahead of print] 30238832

[20] Nakamura J., Meguro A., Ota M., Nomura E., Nishide T., Kashiwagi K. (2009) Association of toll-like receptor 2 gene polymorphisms with normal tension glaucoma. Mol. Vis. 15, 2905–2910 20057905 PMC2802293

[21] Wei X., Cho K.S., Thee E.F., Jager M.J. and Chen D.F. (2019) Neuroinflammation and microglia in glaucoma: time for a paradigm shift. J. Neurosci. Res. 97, 70–76 10.1002/jnr.24256 29775216 PMC6239948

[22] Gomes Torres A., Leite N., Tureck L.V., de Souza R.L.R., Titski A.C.K., Milano-Gai G.E. (2018) Association between Toll-like receptors (TLR) and NOD-like receptor (NLR) polymorphisms and lipid and glucose metabolism. Gene 30481552 10.1016/j.gene.2018.11.065

[23] He B., Xu T., Pan B., Pan Y., Wang X., Dong J. (2018) Polymorphisms of TGFBR1, TLR4 are associated with prognosis of gastric cancer in a Chinese population. Cancer Cell Int. 18, 191 10.1186/s12935-018-0682-0 30479570 PMC6245525

[24] Sghaier I., Zidi S., Mouelhi L., Ghazoueni E., Brochot E., Almawi W.Y. (2018) TLR3 and TLR4 SNP variants in the liver disease resulting from hepatitis B virus and hepatitis C virus infection. Br. J. Biomed. Sci. 76 (1), 35–41 30421643 10.1080/09674845.2018.1547179

[25] Lv Y.B., Wang Y., Ma W.G., Yan D.Y., Zheng W.L., Chu C. (2016) Association of renalase SNPs rs2296545 and rs2576178 with the risk of hypertension: a meta-analysis. PLoS ONE 11, e0158880 10.1371/journal.pone.0158880 27434211 PMC4951046

[26] Martorell-Marugan J., Toro-Dominguez D., Alarcon-Riquelme M.E. and Carmona-Saez P. (2017) MetaGenyo: a web tool for meta-analysis of genetic association studies. BMC Bioinformatics 18, 563 10.1186/s12859-017-1990-4 29246109 PMC5732412

[27] Navarro-Partida J., Martinez-Rizo A.B., Ramirez-Barrera P., Velazquez-Fernandez J.B., Mondragon-Jaimes V.A., Santos-Garcia A. (2017) Association of Toll-like receptor 4 single-nucleotide polymorphisms Asp299Gly and Thr399Ile with the risk of primary open angle glaucoma. Graefe’s archive for clinical and experimental ophthalmology = Albrecht von Graefes Archiv fur klinische und experimentelle. Ophthalmologie 255, 995–1001 10.1007/s00417-017-3610-4 28214954

[28] Navarro-Partida J., Alvarado Castillo B., Martinez-Rizo A.B., Rosales-Diaz R., Velazquez-Fernandez J.B. and Santos A. (2017) Association of single-nucleotide polymorphisms in non-coding regions of the TLR4 gene with primary open angle glaucoma in a Mexican population. Ophthalmic Genet. 38, 325–329 10.1080/13816810.2016.1227454 27892755

[29] Mousa A., Kondkar A.A., Al-Obeidan S.A., Azad T.A., Sultan T., Osman E.A. (2016) Lack of association between polymorphism rs4986791 in TLR4 and primary open-angle glaucoma in a saudi cohort. Genetic Testing Mol. Biomarkers 20, 556–559 10.1089/gtmb.2016.0095 27526043

[30] Abu-Amero K.K., Kondkar A.A., Mousa A., Azad T.A., Sultan T., Osman E.A. (2017) Analysis of toll-like receptor rs4986790 polymorphism in Saudi patients with primary open angle glaucoma. Ophthalmic Genet. 38, 133–137 10.3109/13816810.2016.1151900 27064537

[31] Takano Y., Shi D., Shimizu A., Funayama T., Mashima Y., Yasuda N. (2012) Association of Toll-like receptor 4 gene polymorphisms in Japanese subjects with primary open-angle, normal-tension, and exfoliation glaucoma. Am. J. Ophthalmol. 154, 825.e1–32.e1 10.1016/j.ajo.2012.03.050 22831837

[32] Chen L.J., Tam P.O., Leung D.Y., Fan A.H., Zhang M., Tham C.C. (2012) SNP rs1533428 at 2p16.3 as a marker for late-onset primary open-angle glaucoma. Mol. Vis. 18, 1629–1639 22773901 PMC3388985

[33] Suh W., Kim S., Ki C.S. and Kee C. (2011) Toll-like receptor 4 gene polymorphisms do not associate with normal tension glaucoma in a Korean population. Mol. Vision 17, 2343–2348 21921986 PMC3171501

[34] Shibuya E., Meguro A., Ota M., Kashiwagi K., Mabuchi F., Iijima H. (2008) Association of toll-like receptor 4 gene polymorphisms with normal tension glaucoma. Invest. Ophthalmol. Vis. Sci. 49, 4453–4457 10.1167/iovs.07-1575 18586872

[35] De la Cruz O. and Raska P. (2014) Population structure at different minor allele frequency levels. BMC Proc. 8, S55 10.1186/1753-6561-8-S1-S55 25519390 PMC4143691

[36] Wong J.J., Au A.Y., Ritchie W. and Rasko J.E. (2016) Intron retention in mRNA: No longer nonsense: Known and putative roles of intron retention in normal and disease biology. Bioessays 38, 41–49 10.1002/bies.201500117 26612485

[37] Chaiwiang N. and Poyomtip T. (2019) The associations between Toll-like receptor 4 gene polymorphisms and hepatitis C virus infection: a systematic review and meta-analysis. Biosci. Rep. 39, 10.1042/BSR20182470 30765614 PMC6390129

[38] Guo J., Loke J., Zheng F., Hong F., Yea S., Fukata M. (2009) Functional linkage of cirrhosis-predictive single nucleotide polymorphisms of Toll-like receptor 4 to hepatic stellate cell responses. Hepatology 49, 960–968 10.1002/hep.22697 19085953 PMC2891538

[39] Davoodi H., Hashemi S.R. and Seow H.F. (2013) 5-fluorouracil induce the expression of TLR4 on HCT116 colorectal cancer cell line expressing different variants of TLR4. Iran J. Pharm. Res. 12, 453–460 24250621 PMC3813241

[40] Ohto U., Yamakawa N., Akashi-Takamura S., Miyake K. and Shimizu T. (2012) Structural analyses of human Toll-like receptor 4 polymorphisms D299G and T399I. J. Biol. Chem. 287, 40611–40617 10.1074/jbc.M112.404608 23055527 PMC3504774

[41] Luo C., Yang X., Kain A.D., Powell D.W., Kuehn M.H. and Tezel G. (2010) Glaucomatous tissue stress and the regulation of immune response through glial toll-like receptor signaling. Invest Ophthalmol. Vis. Sci. 51, 5697–5707 10.1167/iovs.10-5407 20538986 PMC3061506

[42] Takeda K. and Akira S. (2005) Toll-like receptors in innate immunity. Int. Immunol. 17, 1–14 10.1093/intimm/dxh186 15585605

[43] Gao W., Xiong Y., Li Q. and Yang H. (2017) Inhibition of toll-like receptor signaling as a promising therapy for inflammatory diseases: a journey from molecular to nano therapeutics. Front. Physiol. 8, 508 10.3389/fphys.2017.00508 28769820 PMC5516312

[44] Lartigue A., Colliou N., Calbo S., Francois A., Jacquot S., Arnoult C. (2009) Critical role of TLR2 and TLR4 in autoantibody production and glomerulonephritis in lpr mutation-induced mouse lupus. J. Immunol. 183, 6207–6216 10.4049/jimmunol.0803219 19841185

[45] Michelsen K.S., Wong M.H., Shah P.K., Zhang W., Yano J., Doherty T.M. (2004) Lack of toll-like receptor 4 or myeloid differentiation factor 88 reduces atherosclerosis and alters plaque phenotype in mice deficient in apolipoprotein E. Proc. Natl Acad. Sci. U.S.A. 101, 10679–10684 10.1073/pnas.0403249101 15249654 PMC489994

[46] Chovanova L., Vlcek M., Krskova K., Penesova A., Radikova Z., Rovensky J. (2013) Increased production of IL-6 and IL-17 in lipopolysaccharide-stimulated peripheral mononuclears from patients with rheumatoid arthritis. Gen. Physiol. Biophys. 32, 395–404 10.4149/gpb_2013043 23817641

[47] Tang C.H., Hsu C.J., Yang W.H. and Fong Y.C. (2010) Lipoteichoic acid enhances IL-6 production in human synovial fibroblasts via TLR2 receptor, PKCdelta and c-Src dependent pathways. Biochem. Pharmacol. 79, 1648–1657 10.1016/j.bcp.2010.01.025 20109438

[48] Lorenz W., Buhrmann C., Mobasheri A., Lueders C. and Shakibaei M. (2013) Bacterial lipopolysaccharides form procollagen-endotoxin complexes that trigger cartilage inflammation and degeneration: implications for the development of rheumatoid arthritis. Arthritis Res. Ther. 15, R111 10.1186/ar4291 24020912 PMC3978890

[49] Sloane J.A., Batt C., Ma Y., Harris Z.M., Trapp B. and Vartanian T. (2010) Hyaluronan blocks oligodendrocyte progenitor maturation and remyelination through TLR2. Proc. Natl Acad. Sci. U.S.A. 107, 11555–11560 10.1073/pnas.1006496107 20534434 PMC2895128

[50] Andersson A., Covacu R., Sunnemark D., Danilov A.I., Dal Bianco A., Khademi M. (2008) Pivotal advance: HMGB1 expression in active lesions of human and experimental multiple sclerosis. J. Leukoc. Biol. 84, 1248–1255 10.1189/jlb.1207844 18644848

[51] Shaw P.J., Barr M.J., Lukens J.R., McGargill M.A., Chi H., Mak T.W. (2011) Signaling via the RIP2 adaptor protein in central nervous system-infiltrating dendritic cells promotes inflammation and autoimmunity. Immunity 34, 75–84 10.1016/j.immuni.2010.12.015 21236705 PMC3057380

[52] Fu Z., Shen Y., Lin L., Chen Y., Li Y. and Que R. (2018) Association between toll-like receptor 4 T399I gene polymorphism and the susceptibility to Crohn’s disease: a meta-analysis of case-control studies. Digestion 97, 250–259 10.1159/000485027 29421805

[53] Smith R.L., Hebert H.L., Massey J., Bowes J., Marzo-Ortega H., Ho P. (2016) Association of toll-like receptor 4 (TLR4) with chronic plaque type psoriasis and psoriatic arthritis. Arch. Dermatol. Res. 308, 201–205 10.1007/s00403-016-1620-4 26830904 PMC4796327

[54] Li T., Jing J., Sun L., Jiang B., Xin S., Yang J. (2018) TLR4 and MMP2 polymorphisms and their associations with cardiovascular risk factors in susceptibility to aortic aneurysmal diseases. Biosci. Rep., 39, 1 10.1042/BSR20181591 PMC6328888 30530865

[55] Jin S.H., Guan X.Y., Liang W.H., Bai G.H. and Liu J.G. (2016) TLR4 polymorphism and periodontitis susceptibility: a meta-analysis. Medicine (Baltimore) 95, e4845 10.1097/MD.0000000000004845 27603404 PMC5023927

[56] Chen H., Cho K.S., Vu T.H.K., Shen C.H., Kaur M., Chen G. (2018) Commensal microflora-induced T cell responses mediate progressive neurodegeneration in glaucoma. Nat. Commun. 9, 3209 10.1038/s41467-018-05681-9 30097565 PMC6086830

[57] Astafurov K., Elhawy E., Ren L., Dong C.Q., Igboin C., Hyman L. (2014) Oral microbiome link to neurodegeneration in glaucoma. PLoS ONE 9, e104416 10.1371/journal.pone.0104416 25180891 PMC4152129

[58] Adornetto A., Russo R. and Parisi V. (2019) Neuroinflammation as a target for glaucoma therapy. Neural. Regen. Res. 14, 391–394 10.4103/1673-5374.245465 30539803 PMC6334605

[59] Williams P.A., Marsh-Armstrong N., Howell G.R. and Lasker IIoA, Glaucomatous Neurodegeneration P (2017) Neuroinflammation in glaucoma: a new opportunity. Exp. Eye Res. 157, 20–27 10.1016/j.exer.2017.02.014 28242160 PMC5497582

[60] Rieck J. (2013) The pathogenesis of glaucoma in the interplay with the immune system. Invest Ophthalmol. Vis. Sci. 54, 2393–2409 10.1167/iovs.12-9781 23539162

[61] Li M., Zhao Y., Yan X. and Zhang H. (2017) The relationship between the 24-hour fluctuations in Schlemm’s Canal and intraocular pressure: an observational study using high-frequency ultrasound biomicroscopy. Current Eye Res. 42, 1389–1395 10.1080/02713683.2017.1324631 28622042

[62] Poyomtip T. (2018) Roles of toll-like receptor 4 for cellular pathogenesis in primary open-angle glaucoma: a potential therapeutic strategy. J. Microbiol. Immunol. Infect. 10.1016/j.jmii.2018.12.006 [In Press] 30612922

