# Supplementary material for: The association of toll-like receptor 4 gene polymorphisms with primary open angle glaucoma susceptibility: a meta-analysis
Source: Biosci Rep. 2019 Apr 2;39(4):BSR20190029. doi: 10.1042/BSR20190029 (PMC6443948; doi:10.1042/BSR20190029)
Supplement: Supplementary file 1 [file bsr20190029_Supp1.pdf]

Funnel plot showed publication bias of rs1927911 G vs. A

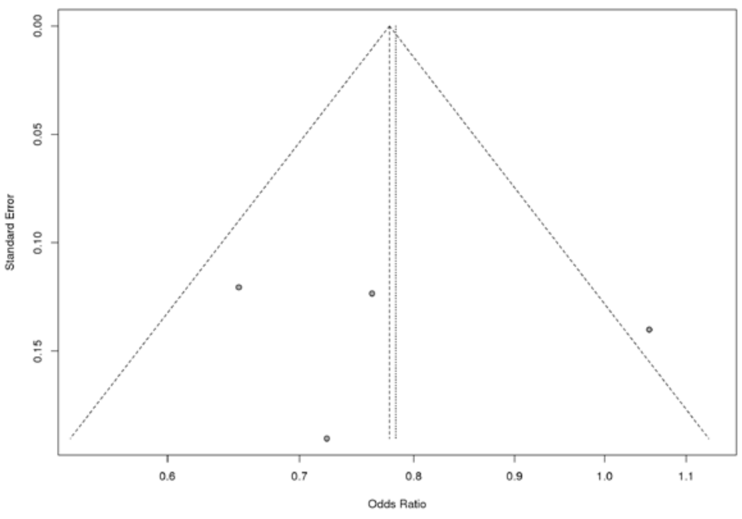

Funnel plot showed publication bias of rs12377632 C vs. T

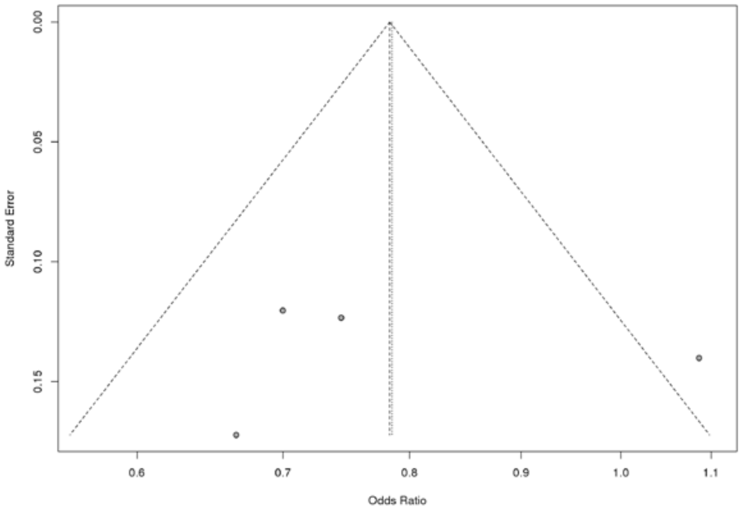

Funnel plot showed publication bias rs2149356 G vs. T

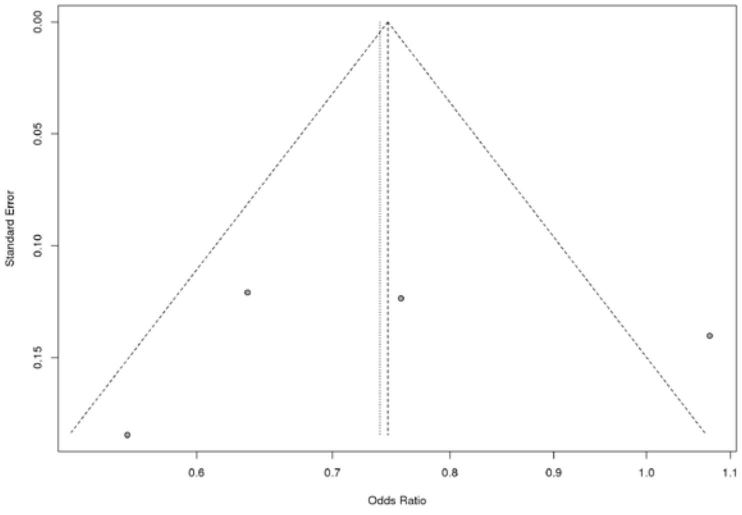

Supplementary Table 1 The NOS analysis of inclued studies

Study ID	Navarro-Partida et al. 2017b	Navarro-Partida et al. 2017a	Mousa et al. 2016	Abu-Amero et al. 2017	Takano et al. 2012	Chen et al. 2012	Suh et al. 2011	Shibuya et al. 2008
**Selection**								
Is the case definition adequate?	Yes (a*)	Yes (a*)	Yes (a*)	Yes (a*)	Yes (a*)	Yes (a*)	Yes (a*)	Yes (a*)
Representativeness of the cases	Not stated	Not stated	Not stated	Not stated	Not stated	Not stated	consecutive cases (a*	Not stated
Selection of Controls	community controls and genetic polymophisms of interest in HWE (a*)	Not stated	hospital controls and genetic polymophisms of interest in HWE	hospital controls and genetic polymophisms of interest in HWE	Not stated	hospital controls and genetic polymophisms of interest in HWE	Not stated	community controls and genetic polymophisms of interest in HWE (a*)
Definition of Controls	Yes (a*)	Yes (a*)	Yes (a*)	Yes (a*)	Yes (a*)	Yes (a*)	Yes (a*)	Yes (a*)
**Comparability**								
cases and controls of homogeneous ethnic descent	may heterogenous population between cases and control	may heterogenous population between cases and control	may heterogenous population between cases and control	may heterogenous population between cases and control	may heterogenous population between cases and control	Homogenous ethinic descent (a*): Han Chinese	Homogenous ethinic descent (a*): Korean ethnicity	may heterogenous population between cases and control
no evidence of population stratification	Maxican population (a*)	Maxican population (a*)	Saudi population (a*)	Saudi population (a*)	Japanese Population (a*)	Chinese population (a*)	South Kprean population (a*)	Japanese Population (a*)
**Exposure**								
Ascertainment of exposure	no report of quality control precedures or blinding	no report of quality control precedures or blinding	no report of quality control precedures or blinding	no report of quality control precedures or blinding	triplicated samples and duplicate analysis but not report blinding	no report of quality control precedures or blinding	no report of quality control precedures or blinding	no report of quality control precedures or blinding
Same method of ascertainment for cases and controls	Yes (a*)	Yes (a*)	Yes (a*)	Yes (a*)	Yes (a*)	Yes (a*)	Yes (a*)	Yes (a*)
Genotyping call rate	Not reported	Not reported	Not reported	Not reported	Not reported	Not reported	Yes (a*)	Not reported
Total	5	4	4	4	4	5	6	5

Supplement Table 2 The Egger's test showed publication bias of meta-analysis in Table 3

SNPs	Genetic Models						
	A	B	C	D	E	F	G
rs4987690 A/G	ND	ND	ND	ND	ND	ND	ND
rs4987691 C/T	ND	ND	ND	ND	ND	ND	ND
rs10759930 T/C	0.01	0.17	0.21	0.09	0.11	0.13	0.00
rs1927914 A/G	0.03	0.17	0.79	0.04	0.24	0.04	0.18
rs1927911 G/A	0.82	0.50	0.02	0.96	0.32	0.76	0.68
rs12377632 C/T	0.95	0.87	0.93	0.91	0.52	0.99	0.31
rs2149356 G/T	0.82	0.98	0.08	0.72	0.77	0.84	0.49
rs11536889 G/C	0.66	0.32	0.17	0.83	0.26	0.75	0.81
rs7037117 A/G	0.16	0.12	0.15	0.00	0.02	0.02	0.01
rs7045953 A/G	0.39	0.21	0.43	0.43	0.24	0.38	0.44

ND: Non-detemined

A: Allele contrast model, B: Homozygous model, C: Heterozygous (12 vs 22), D: Heterozygous (11 vs 12), E: Dominant model, F: Recessive model, and G: Overdominant model
